# Insulin mediates *de novo* nuclear accumulation of the IGF-1/insulin Hybrid Receptor in corneal epithelial cells

**DOI:** 10.1038/s41598-018-21031-7

**Published:** 2018-03-12

**Authors:** Rossella Titone, Meifang Zhu, Danielle M. Robertson

**Affiliations:** The Department of Ophthalmology, The University of Texas Southwestern Medical Center, Dallas, Texas USA

## Abstract

Insulin and insulin-like growth factor-1 (IGF-1) are present in human tears and likely play an important role in mediating ocular surface homeostasis. We previously characterized the IGF-1/insulin hybrid receptor (Hybrid–R) in corneal epithelial cells and found that it was activated by IGF-1 and not insulin; and reported the novel finding that it localized to the corneal epithelial cell nucleus. Since the corneal epithelium is an insulin insensitive tissue and does not require insulin for glucose uptake, this study investigated the function of insulin in corneal epithelial cells. We show that stress induced by growth factor deprivation triggers transcriptional upregulation and *de novo* nuclear accumulation of Hybrid-R through the homodimeric insulin receptor (INSR). This occurs independent of PI3K/Akt signaling. Nuclear accumulation of Hybrid-R was associated with partial cell cycle arrest and a corresponding reduction in mitochondrial respiration. Treatment with insulin, and not IGF-1, attenuated IGF-1R and INSR transcription and restored cell cycle and metabolic homeostasis. Together, these findings support that insulin mediates receptor homeostasis in corneal epithelial cells, favoring an IGF-1 mediated pathway. This may have important implications in diabetic corneal disease and wound healing.

## Introduction

Insulin Receptor (INSR) and Insulin-like Growth Factor Type 1 Receptor (IGF-1R) are members of the receptor tyrosine kinase superfamily^1^. They play an important role in the regulation of essential biological and molecular processes including proliferation, migration, metabolism, differentiation, and survival^2^. This occurs through ligand binding of the receptor at the plasma membrane, leading to autophosphorylation and downstream activation of phosphoinositide 3-kinase (PI3K) and extracellular signal regulated kinase (ERK) pathways^3^. Known extracellular ligands for INSR and IGF-1R include insulin, IGF-1, and IGF-2, all of which display different affinities for each receptor^1^.

Structurally, INSR and IGF-1R are transmembrane glycoproteins composed of two extracellular alpha subunits that form the ligand-binding domain and two transmembrane beta subunits that possess tyrosine kinase activity^4^. Overall, the two receptors exhibit greater than 50% homology in their amino acid sequences. This ranges from 45% to 65% in the alpha subunit binding domain, rising to 84% homology within the tyrosine kinase domain. The structural similarity between INSR and IGF-1R make possible the formation of insulin and IGF-1 hybrid receptors (Hybrid-R)^5–7^. It is unknown what drives formation of Hybrid-R. Some hypothesize that formation of Hybrid-R is driven by the ratio between IGF-1R and INSR^8^. Others speculate that Hybrid-R is regulated developmentally^9^. In addition to formation of Hybrid-R, the functional significance of Hybrid-R remains controversial. An increase in Hybrid-R expression has been reported in skeletal muscle and adipose tissue in diabetes^10–12^. Hybrid-R has also been shown to bind IGF-1 with a greater affinity than insulin^13,14^. Thus, increased expression of Hybrid-R in diabetic tissue may alter insulin sensitivity^7,10–12^. A reduction in insulin sensitivity represents a key hallmark of diabetes.

In the diabetic cornea, epithelial erosions, persistent epithelial defects, corneal neuropathy and ulceration can result in painful and often permanent loss of vision^15–18^. While the corneal epithelium has been previously reported to be an insulin-insensitive tissue, meaning that it does not require insulin for glucose uptake, studies have shown that supraphysiological levels of insulin applied topically to the eye promotes corneal wound healing in animals with diabetes^19,20^. Our prior work has confirmed that the IGF-system is altered in diabetic tears^21^. We have further demonstrated the presence of Hybrid-R in human corneal epithelial cells and shown that Hybrid-R was preferentially expressed over either homodimeric receptor^8^. Interestingly, we found that Hybrid-R, but not homodimeric IGF-1R, is present in the corneal epithelial cell nucleus. Prior studies have shown IGF-1-mediated translocation of IGF-1R to the nucleus in embryonic and cancer cells. In this study however, we show that accumulation of Hybrid-R in the corneal epithelial cell nucleus is not mediated by IGF-1 binding at the plasma membrane, but occurs *de novo* in response to stress induced by growth factor deprivation^22,23^. We further found that nuclear accumulation is associated with partial cell cycle arrest and a reduction in mitochondrial respiration. This is restored upon treatment with insulin and occurs via the homodimeric INSR. Thus, in the cornea, Hybrid-R expression is mediated by the presence of insulin and serves to regulate key functions required for cell growth and survival.

## Results

### Upregulation of IGF-1R and INSR in basal medium

**Figure 1:**
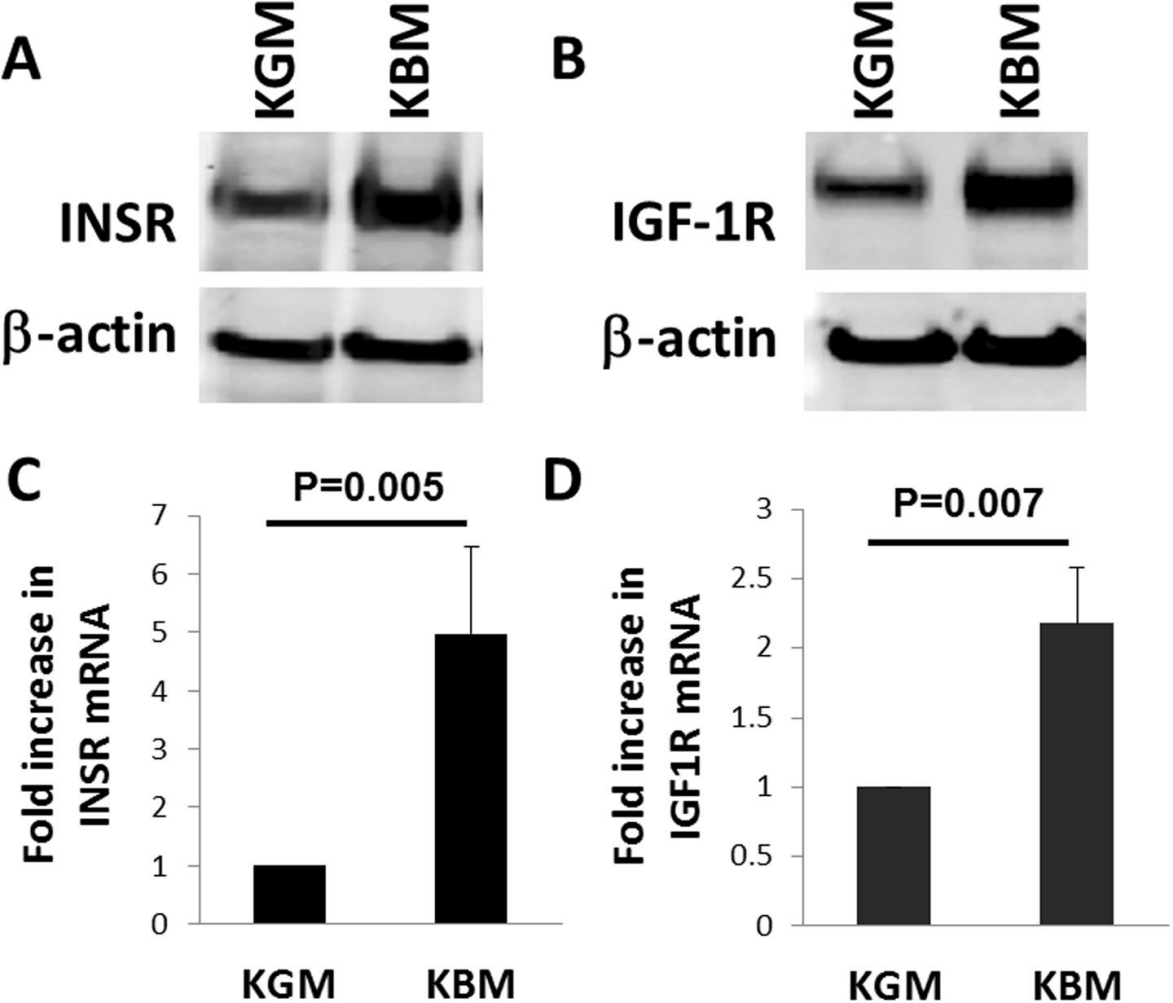
Growth factor deprivation upregulates IGF-1R and INSR. hTCEpi cells were cultured overnight in growth or basal media. (**A**) Western blotting for INSR showed an increase in INSR expression in basal media. (**B**) Similar to INSR, IGF-1R also showed an increase in expression in basal media compared to growth conditions. β-actin was used as a loading control. (**C**) Real time PCR for INSR in hTCEpi cells showed a significant upregulation of INSR mRNA in basal media compared to growth (P = 0.005, t-test). (**D**) Real time PCR for IGF-1R also showed a significant upregulation of IGF-1R mRNA in basal media (P = 0.007, t-test). Real time PCR data normalized against β-actin and calculated using the 2- ΔΔCT method. Data represented as mean ± standard deviation of fold change in expression in basal media (KBM) compared to growth (KGM).

In our prior studies, growth factor withdrawal failed to deplete IGF-1R from the nucleus of corneal epithelial cells^24^. To evaluate overall expression levels of INSR and IGF-1R in response to growth factor deprivation, cells were cultured in growth (KGM, containing supplements) and basal (KBM, devoid of supplements) media for 24 hours. Expression levels of INSR and IGF-1R were assessed by immunoblotting. Compared to culture in growth media, there was a large increase in the expression of both INSR and IGF-1R when cultured in basal conditions (Fig. 1A and B, respectively). Real time PCR for INSR and IGF-1R confirmed that mRNA levels of INSR (P = 0.005, Student’s t-test) and IGF-1R (P = 0.007, Student’s t-test) were significantly upregulated when cultured in basal media (Fig. 1C and D).

### De novo nuclear accumulation of IGF-1/insulin Hybrid-R

**Figure 2:**
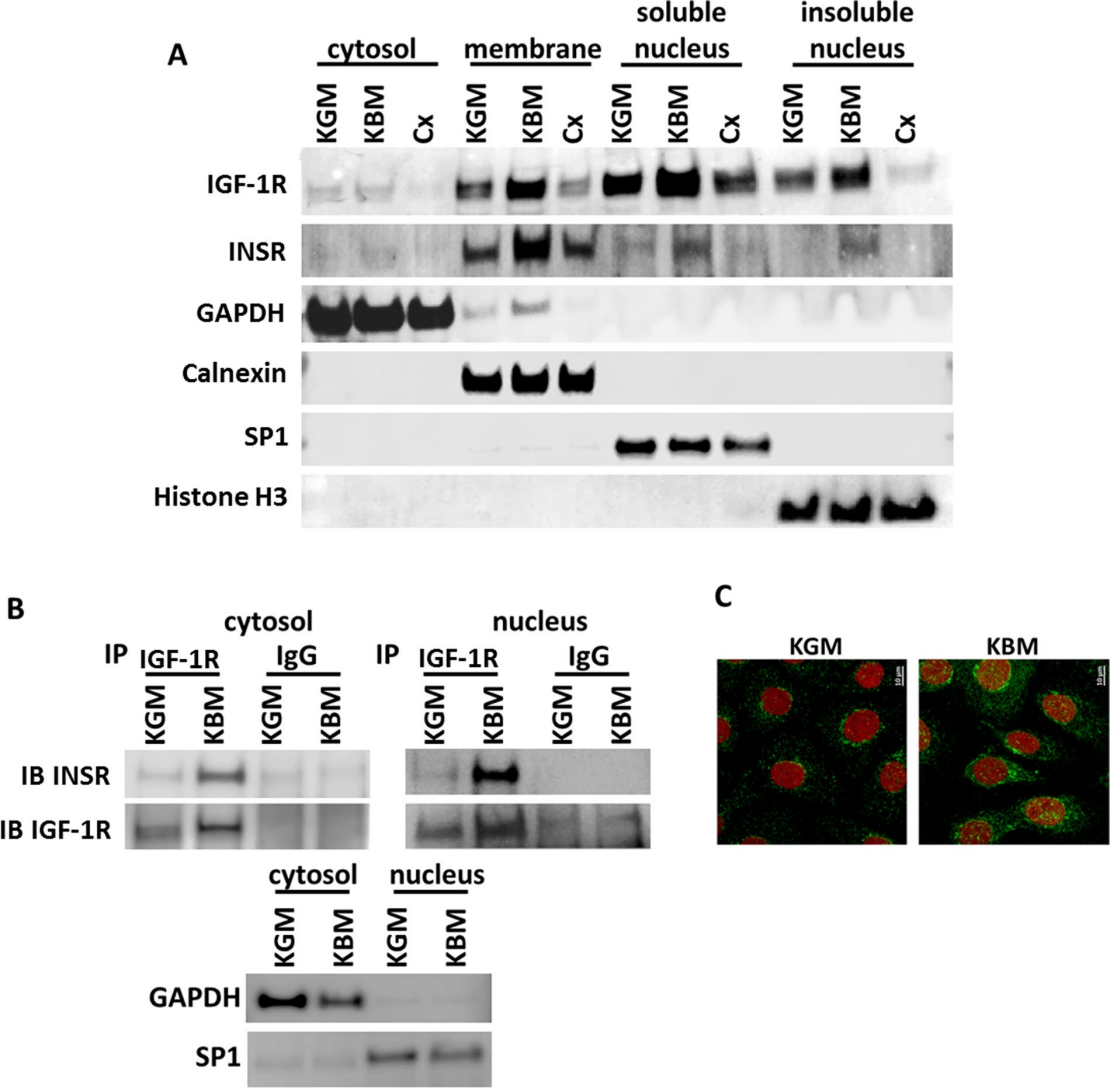
Nuclear accumulation of the IGF-1/insulin Hybrid R occurs *de novo* in response to growth factor deprivation. (**A**) hTCEpi cells were cultured in growth, basal or basal media supplemented with 1 μg/ml cycloheximide (Cx) overnight. Cells were lysed and fractionated into cytosolic, membrane, soluble and insoluble nuclear fractions. Consistent with whole cell lysates, immunoblotting for IGF-1R showed an increase in IGF-1R expression in basal media compared to controls. Treatment with cycloheximide blunted the basal increase and reduced expression levels to below those seen in growth media. There was a large increase in nuclear accumulation of IGF-1R in both the soluble and insoluble fractions. Similar to IGF-1R, INSR was also increased in basal media. Cycloheximide treatment of samples in basal media blocked nuclear accumulation of INSR. Immunoblotting for GAPDH, Calnexin, SP1 and Histone H3 were used for cytosolic, membrane, soluble nucleus, and insoluble nucleus fractionation controls. (**B**) hTCEpi cells were cultured overnight in basal or growth media and fractionated into nuclear and non-nuclear (cytosolic) components. Samples were immunoprecipated for IGF-1R and immunoblotted for INSR and IGF-1R to confirm the presence of nuclear Hybrid-R. There was an increase in Hybrid-R expression in the nuclear compartment in basal media. GAPDH and SP1 were used for cytosolic and nuclear fraction contamination controls. (**C**) Immunofluorescence for IGF-1R (green) and nuclei (red) shows an increase in IGF-1R expression and nuclear accumulation in basal media compared to growth media.

To determine the subcellular localization of IGF-1R and INSR in corneal epithelial cells, cells were cultured in growth media, basal media, or basal media supplemented with 1 μg/ml cycloheximide overnight to inhibit protein translation. Fractionation of the individual cellular components revealed the presence of both receptors in the membrane fraction and the soluble and insoluble nuclear fractions (Fig. 2A). There was an increase in nuclear accumulation of IGF-1R and INSR in basal conditions in all fractions that was abrogated following treatment with cycloheximide. Loading controls, GAPDH, Calnexin, SP1, and Histone H3 for cytoplasm, membrane, soluble and insoluble nuclear fractions, respectively, confirmed successful separation of individual fractions. To confirm that increased receptor expression in the nucleus was Hybrid-R, hTCEpi cell nuclear and non-nuclear (cytosolic) fractions were immunoprecipitated with an antibody recognizing the beta subunit of IGF-1R and immunoblotted for INSR and IGF-1R. In both cytoplasmic and nuclear fractions, there was an increase in the presence of Hybrid-R under basal conditions (Fig. 2B). Expression of Hybrid-R was greater in the nuclear fraction. To further demonstrate nuclear accumulation of IGF-1R and to rule out any effects of trypsinization on the cell fractionation studies, hTCEpi cells were cultured in growth and basal media and stained for immunofluorescence using an antibody directed against the beta subunit of IGF-1R. Nuclei were counterstained with DAPI. Consistent with immunoblotting, in basal conditions, there was an increase in the intensity of IGF-1R fluorescence and a large increase in nuclear localization (Fig. 2C).

### Insulin regulates IGF-1R expression through the homodimeric INSR

**Figure 3:**
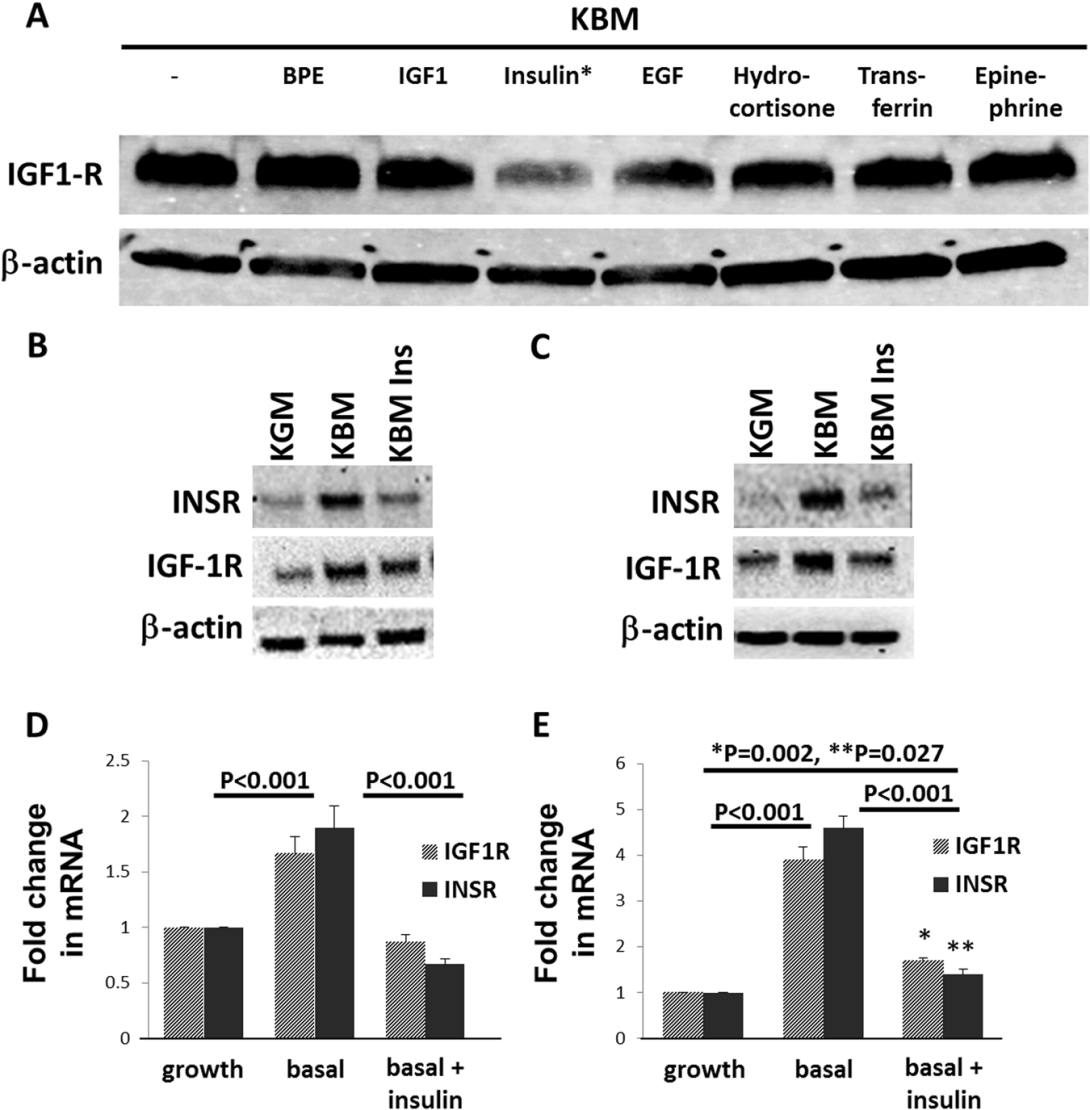
Insulin treatment in basal media restores IGF-1R and INSR to normal levels. (**A**) hTCEpi cells were cultured in basal media and supplemented individually with each component of the bullet kit (BPE, hEGF, insulin, hydrocortisone, transferrin, epinephrine) and also with 100 ng/ml of IGF-1. Expression of IGF-1R was assessed by immunoblotting. Insulin, a component of the bullet kit, was the only growth factor that decreased expression of IGF-1R. **(B**) hTCEpi cells were cultured in growth, basal or basal media supplemented with 10 μg/ml insulin. For both receptors, immunoblotting showed an increase in basal media that was attenuated following treatment with insulin. (**C**) HCECs were also cultured overnight in growth, basal or basal media supplemented with 10 μg/ml insulin. Similar to hTCEpi cells, there was a large increase in INSR and IGF-1R expression in basal media that was reduced following the addition of insulin to the culture media. (**D**) Real time PCR for IGF-1R and INSR in hTCEpi cells showed that changes in receptor expression levels paralleled mRNA levels with a significant increase in IGF-1R and INSR mRNA in basal media compared to growth media or basal media supplemented with insulin (P < 0.001, Two-way ANOVA, Tukey multiple comparison test). (**E**) Real time PCR for IGF-1R and INSR in HCECs. There was a significant increase in IGF-1R and INSR in basal media compared to growth conditions or basal media supplemented with insulin (P < 0.001, Two-way ANOVA, Tukey multiple comparison test). There was a small, but significant difference between IGF-1R and INSR mRNA in basal media supplemented with insulin compared to the growth condition (*P = 0.002 for IGF-1R and **P = 0.027 for INSR, Two-way ANOVA, Tukey multiple comparison test). Data represented as mean ± standard deviation of fold change compared to growth condition.

To investigate whether a specific growth factor or hormone present in growth media was responsible for regulating IGF-1R expression, hTCEpi cells were cultured for 24 hours in basal media supplemented with each individual growth factor present in the bullet kit. These include bovine pituitary extract, human epidermal growth factor, insulin, hydrocortisone, transferrin, and epinephrine. IGF-1, which is present in trace amounts in bovine pituitary extract, but not present as a stand-alone supplement, was also tested at a concentration of 100 ng/ml. IGF-1R was abundant in all samples tested, except for cells exposed to insulin (Fig. 3A). Immunoblotting and real time PCR for INSR and IGF-1R in basal media with and without insulin supplementation confirmed that the changes in receptor expression paralleled transcriptional effects (Fig. 3B,D, respectively). Importantly, treatment with insulin restored mRNA and protein levels for each receptor back to normal growth levels. To confirm that this response was not a result of a cell line artefact, we tested HCECs cultured in growth media, basal media, and basal media supplemented with insulin. The pattern of response of HCECs to growth factor deprivation and insulin treatment paralleled the response seen in the hTCEpi cell line, although the response was greater in magnitude (Fig. 3C,E). HCECs showed a 4 fold upregulation in IGF-1R and INSR in response to growth factor deprivation while hTCEpi cells were only increased 1.5–2 fold.

**Figure 4:**
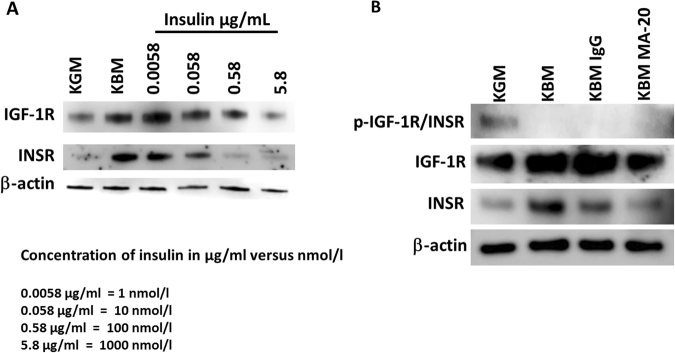
Growth factor deprivation-mediated increase in IGF-1R and INSR is not regulated by phosphorylation of the homodimeric INSR. (**A**) hTCEpi cells were cultured in growth or basal media and treated with 1, 10, 100, 1000 nmol/l of insulin. At the physiological concentrations (1 nmol/l), treatment with insulin reduced INSR expression but increased IGF-1R expression levels. Treatments with increasing concentrations of insulin from 10 nmol/l up to 1000 nmol/l showed a decrease in both IGF-1R and INSR levels. (**B**) hTCEpi cells were cultured in growth or basal media. For cells in basal media, cells were treated with the insulin mimic MA-20 or an irrelevant IgG control. Treatment with MA-20 did not induce phosphorylation of INSR/IGF-1R, but did reduce expression of IGF-1R and INSR back to growth levels.

To assess the effects of insulin concentration on receptor expression, hTCEpi cells were treated with physiological and supraphysiological concentrations of insulin (Fig. 4A,B). At the low end of physiological concentrations (1 nmol/l), treatment with insulin reduced INSR expression while IGF-1R expression levels were increased. The addition of increasing levels of insulin from 10 nmol/l and up showed a downregulation of both IGF-1R and INSR levels at all concentrations tested. Importantly, from 10 nmol/l and up, this effect was dose dependent, until reaching a saturating supraphysiological level (Fig. 4A and Supplementary Figure 1).

**Figure 5:**
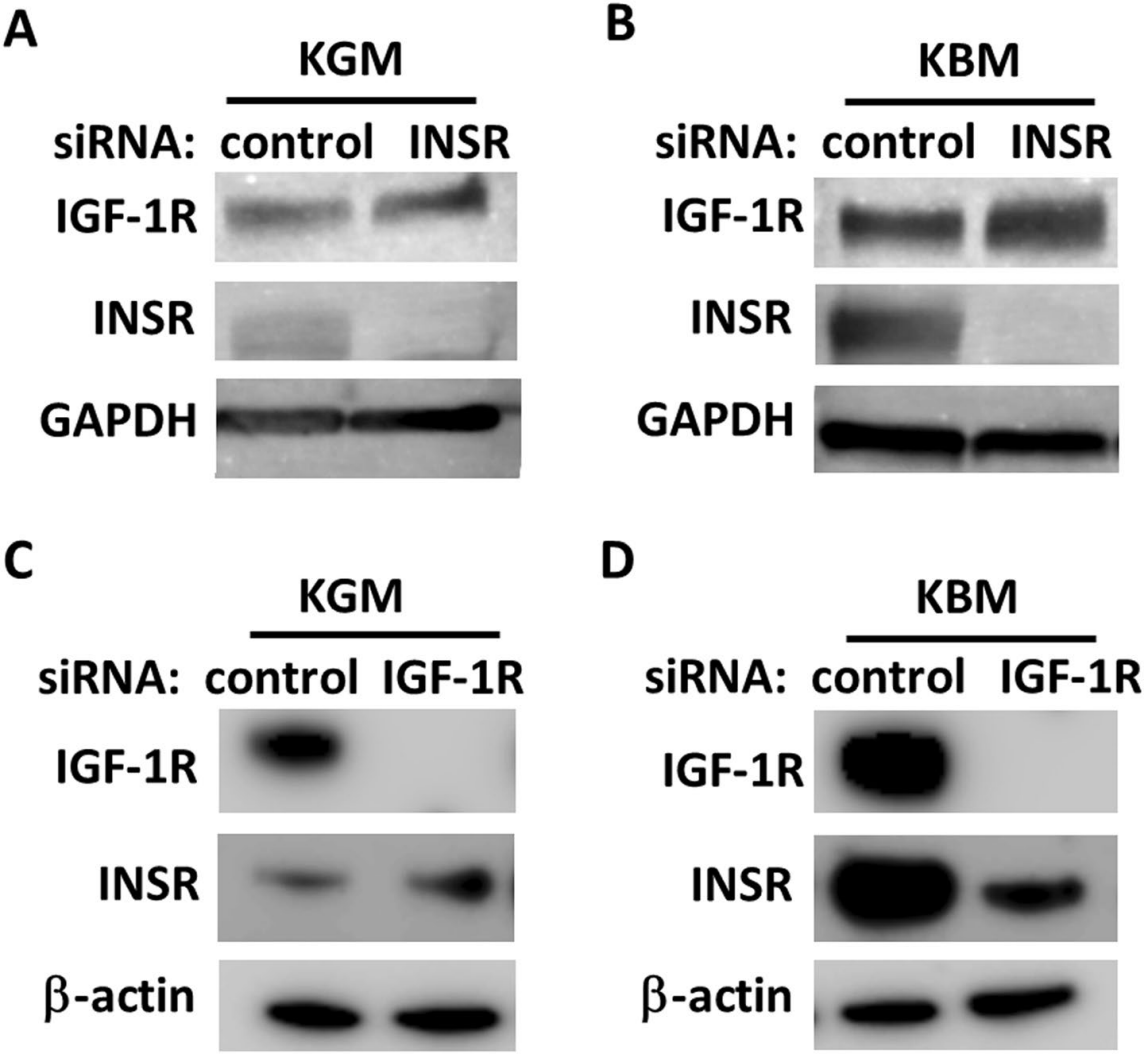
Homodimeric INSR controls IGF-1R expression. (**A**) hTCEpi cells treated with siRNA oligonucleotides targeting the INSR resulted in a small increase in IGF-1R in growth media. (**B**) In contrast, siRNA knockdown of INSR in basal media induced a large increase in IGF-1R expression compared to the non-targeting control. Immunoblotting for INSR confirmed knockdown of the INSR. GAPDH was used as a loading control. (**C**) Similarly, siRNA knockdown of IGF-1R in growth media led to a small increase in INSR in growth media. (**D**) However, siRNA knockdown of IGF-1R in basal media led to a major reduction in INSR levels. Immunoblotting for IGF-1R confirmed knockdown of the receptor. β-actin was used as a loading control.

To investigate whether insulin regulated IGF-1R expression through the INSR, we first blocked the homodimeric INSR using a monoclonal antibody, clone MA-20, that recognized the extracellular alpha-subunit of the homodimeric INSR. Treatment with MA-20 in basal media reduced expression of both IGF-1R and INSR compared to the IgG control (Fig. 4B). Moreover, the presence of MA-20 in basal media restored IGF-1R expression levels back to the normal, growth control. Treatment with MA-20 did not induce measurable levels of phosphorylation of IGF-1R or INSR. To confirm the role of the homodimeric INSR in regulating IGF-1R, INSR expression was knocked down using siRNA. Our previous data has shown that siRNA knockdown of INSR preferentially decreases the homodimeric INSR and not the IGF-1/insulin Hybrid-R^8^. In growth media, knockdown of INSR resulted in only a slight increase in IGF-1R (Fig. 5A), whereas in basal media, knockdown of INSR triggered a large increase in IGF-1R (Fig. 5B). In contrast, a slight increase in INSR was seen following IGF-1R knockdown in growth media (Fig. 5C). Knockdown of IGF-1R in basal media did not have the same effect however, and resulted in a corresponding reduction INSR (Fig. 5D).

### Treatment with insulin in basal media induces phosphorylation of Akt in hTCEpi cells

To determine whether insulin initiated PI3K/Akt signalling mediated expression of IGF-1R or INSR, cells were treated with the PI3 kinase inhibitor LY 294002 (Supplementary Fig. 2). Treatment with LY294002 did not block insulin-induced phosphorylation of INSR, but did block phosphorylation of Akt. Compared to the DMSO vehicle control, blocking PI3K/Akt signalling did not alter expression of IGF-1R or INSR.

### Nuclear translocation of the IGF-1/insulin Hybrid-R is associated with cell cycle arrest and reduced oxygen consumption

**Figure 6:**
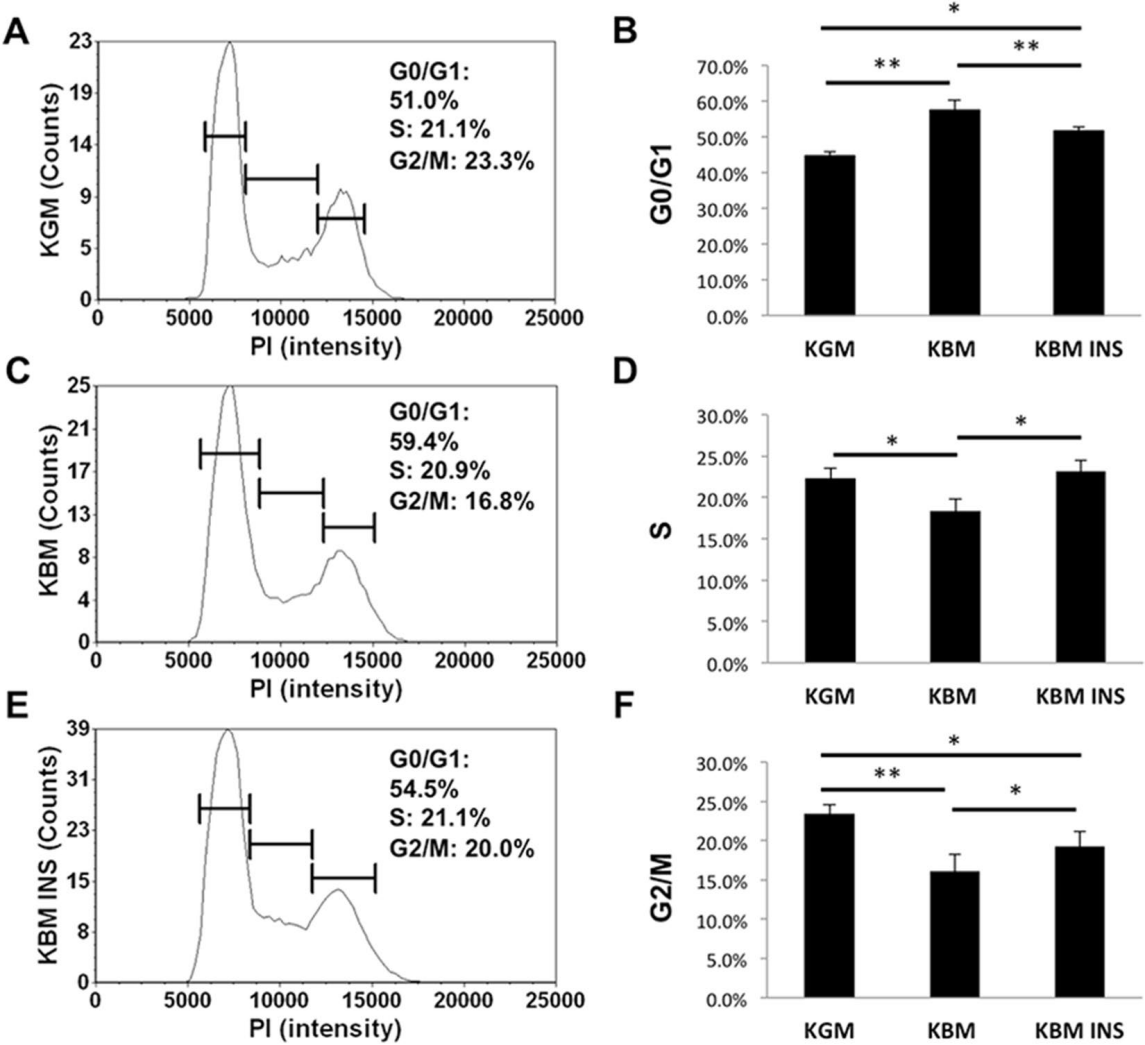
Growth factor deprivation induces a partial cell cycle arrest that is restored by insulin. (**A**, **C** and **E**) Representative graphs of the cell count as a function of PI intensity in (**A**) growth media; (**C**) basal media; (**E**) basal media supplemented with insulin. (**B**) Cells cultured in basal media showed an increase in the proportion of cells in G0/G1 compared to growth conditions (**P < 0.001, One-way ANOVA, Holm-Sidak multiple comparison test). This was restored by insulin (basal versus basal + insulin, **P < 0.001; growth versus basal + insulin, *P < 0.05, One-way ANOVA, Holm-Sidak multiple comparison test). Data represented as mean ± standard deviation.

**Figure 7:**
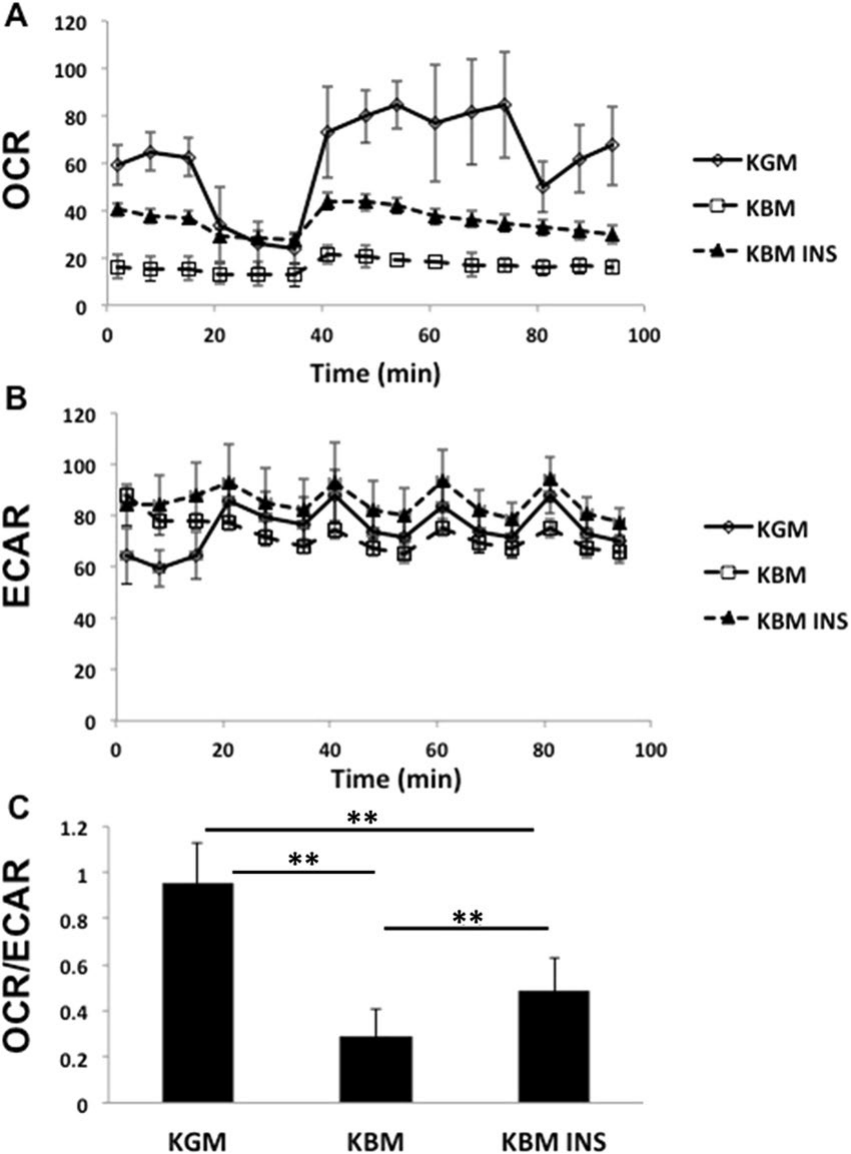
Insulin increases mitochondrial respiration in corneal epithelial cells. (**A**) Oxygen consumption rate (OCR) in hTCEpi cells as a function of time. KBM reduces cellular oxygen uptake in basal conditions and is partially restored by insulin. (**B**) Extracellular acidification rate (ECAR) is relatively unchanged during growth factor deprivation. (**C**) Ratio of OCR/ECAR as an indicator of glycolytic activity. There is a statistical decrease in mitochondrial respiration, shifting cells to a higher glycolytic state in basal media (**P < 0.001, One-Way ANOVA, Holm-Sidak multiple comparison test). Treatment with insulin partially restores mitochondrial homeostasis by increasing mitochondrial respiration (**P < 0.001, One-Way ANOVA, Holm-Sidak multiple comparison test). Data represented as mean ± standard deviation. Dotted vertical lines indicate the timing of injection of oligomycin (OM) and carbonyl cyanide 4-(trifluoromethoxy) phenylhydrazone (FCCP).

To examine the physiological effect of growth factor deprivation on cell cycle control, hTCEpi cells were stained with propidium iodide and analysed using a Cellometer. As expected, in basal media, there was a significant increase in cells arrested in G_0_/G_1_ compared to cells cultured in growth media (Fig. 6A and B). This was associated with a reduction in cells in S phase and in G_2_/M (Fig. 6C,D). Cell cycle was restored following treatment with insulin. Similar to the cell cycle, there was a corresponding reduction in oxygen consumption in basal media (Fig. 7A,C). The basal oxygen consumption rate for cells in growth media was nearly 4 fold higher than cells in basal media, but only 1.5 fold higher than basal media supplemented with insulin (Fig. 7A). The addition of oligomycin to block ATP production resulted in a characteristic drop in oxygen consumption for hTCEpi cells in growth conditions and a similar, albeit less dramatic drop in hTCEpi cells cultured in basal media with insulin. hTCEpi cells cultured in basal media alone were unaffected by the addition of oligomycin, indicating that the cells were not reliant on oxygen-dependent production of ATP. The subsequent addition of the electron transport chain uncoupler FCCP revealed the highest maximum respiratory capacity for cells cultured in growth media, followed by cells cultured in basal media containing insulin. hTCEpi cells cultured in basal media were relatively unchanged.

ECAR, which is a robust measure of glycolysis, was increased in hTCEpi cells cultured in growth media after addition of oligomycin, to compensate for the drop in ATP production (Fig. 7B). There was a slight, partial increase in ECAR in hTCEpi cells cultured in basal media with insulin; however, cells cultured in basal media alone were unchanged. Calculation of the OCR/ECAR ratio at baseline revealed that cells cultured in growth media were highly glycolytic and ECAR was not impacted by any residual CO_2_ from the mitochondria (Fig. 7C). In basal media, there was a 70% decrease (**P < 0.001, One-way ANOVA) in the OCR/ECAR ratio indicating a shift toward a more highly glycolytic state. Treatment with insulin partially restored mitochondrial respiration in hTCEpi cells (**P < 0.001, One-way ANOVA).

## Discussion

In this study, we show for the first time, *de novo* nuclear accumulation of the IGF-1/insulin Hybrid-R. In contrast to highly proliferative non-malignant and malignant cells in which IGF-1 triggers translocation of IGF-1R from the plasma membrane to the nucleus, nuclear accumulation of Hybrid-R occurs independent of ligand binding^23,25,26^. Similarly, where nuclear IGF-1R has been shown to be present in invasive tumors, nuclear accumulation of Hybrid-R in corneal epithelial cells occurs in response to stress-induced growth factor deprivation and is associated with a partial cell cycle arrest and a corresponding reduction in cellular respiration^22,23^. The decrease in cellular respiration shifted hTCEpi cells to a more highly glycolytic state, as shown by the decrease in the OCR/ECAR ratio. Corneal epithelial cells are highly glycolytic in nature and possess a very high level of glycogen, thus, stress-induced growth factor deprivation readily increases glycolysis in corneal epithelial cells to compensate for the reduction in mitochondrial respiration^27,28^. Future studies are necessary to understand the molecular mechanisms that regulate metabolic and mitochondrial respiration in corneal epithelial cells.

The function of insulin in corneal epithelial cells is controversial. The corneal epithelium has been shown to be an insulin-insensitive tissue in that insulin is not required for glucose uptake^29^. In culture, insulin promotes proliferation and migration of corneal epithelial cells; however, the mechanism for this is not well defined^30,31^. Insulin has also been shown to restore circadian rhythm and diurnal control of proliferation in type 1 streptozotocin-induced diabetic mice^32^. Here we show that insulin restores the cell cycle and regulates mitochondrial homeostasis. This occurs through the homodimeric INSR. This finding is supported by use of the insulin mimic MA-20, which we have previously shown binds specifically to the homodimeric INSR and not Hybrid-R^8^.

Interestingly, there appears to be a novel linkage between IGF-1R and INSR. We have previously shown that knockdown of INSR primarily decreases expression of the homodimeric INSR and not Hybrid-R. Moreover, siRNA knockdown of INSR fails to deplete INSR from the nucleus^8^. Instead, knockdown of INSR and blockade of the homodimeric INSR boosts expression of IGF-1R in corneal epithelial cells. In contrast to this, INSR is not increased following knockdown of IGF-1R, but instead is decreased in basal media. Our prior work has shown that Hybrid-R, like IGF-1R, responds primarily to IGF-1 and shows a weaker response to insulin when regulating proliferation in corneal epithelial cells^8,13^. This is consistent with other reports of Hybrid-R demonstrating a higher affinity towards IGF-1 than insulin. Together, these data indicate that corneal epithelial cells favor an IGF-1-mediated pathway and the presence of extracellular insulin plays an important role in regulating receptor homeostasis.

It is important to note, that most of the studies performed in this report included supraphysiological levels of insulin. At the low end of the normal physiological threshold, 1 nmol/l of insulin added after a 24 hour starvation period was able to decrease INSR, yet IGF-1R was increased. We speculate this response is due to the increase in receptor levels following 24 hours of culture in basal medium, increasing sensitivity to insulin. Similarly, knockdown of INSR also boosted IGF-1R levels, suggesting that IGF-1R is likely increasing to compensate for low levels of INSR. In stark contrast to this, increasing concentration levels of insulin within the physiological range decreased expression of both receptors in a dose dependent manner. We posit that this occurs in response to high levels of ligand driving receptor levels down to control for unwanted cell growth. Moreover, upon reaching supraphysiological levels, the effects of insulin plateau. We chose to use supraphysiological levels of insulin in this study in order to simulate the magnitude of change seen in receptor expression between growth and basal conditions. Importantly, this high level of insulin added to basal media was required to restore expression of both receptors back to normal growth levels.

While these studies were performed *in vitro* and shed light on the mechanism in which insulin regulates IGF-1R and INSR expression and localization during stress, supraphysiological concentrations have also been shown to be beneficial *in vivo*. In the diabetic cornea, topical application of high levels of exogenous insulin has been shown to promote wound healing^20,33^. Insulin has been reported to be present in human tears and is stored and secreted from the lacrimal gland^34–36^. In diabetes, the progressive loss of the subbasal nerve plexus, combined with hyperglycaemic and oxidative damage to the lacrimal gland, likely drive a reduction in essential trophic factors, including insulin, that are secreted into the diabetic tear film. Likewise, the Hybrid-R, which is increased in diabetic tissues, is thought to play a role in mediating insulin sensitivity^7,10–12^. The potential for a drop in tear levels of insulin in diabetes and a corresponding increase in nuclear Hybrid-R may contribute to abnormal corneal epithelial homeostasis and metabolism. This may account for the delayed resurfacing of recurrent corneal erosions and persistent corneal epithelial defects through the downregulation of proliferation and a drop in cellular energy production. Further studies are necessary to explore these findings.

The finding that growth factor deprivation upregulates IGF-1R and INSR and drives *de novo* nuclear accumulation of Hybrid-R suggests that Hybrid-R plays an important role in the corneal epithelial stress response. Since IGF-1R and INSR were both shown to localize to the insoluble nuclear fraction, this suggests a role in gene regulation^8,24^. This is consistent with our prior ChIP-seq findings^8^. Likewise, Warsito and colleagues have shown that IGF-1R functions as a transcriptional co-activator and promotes levels of Cyclin D1^37^. Unlike cancer cells, corneal epithelial cells are non-invasive and growth down-regulated. Based on our findings, we hypothesize that levels of Hybrid-R increase in the nucleus as part of the corneal epithelial stress response to promote survival of the corneal epithelium; whereas highly proliferative cells that contain high levels of homodimeric IGF-1R, use an alternative IGF-1 driven pathway to stimulate proliferation and invasiveness^23,26,38,39^. This implicates Hybrid-R as an important mediator of corneal epithelial homeostasis in the normal and diseased eye and studies are on-going to identify nuclear targets for Hybrid-R in the corneal epithelium.

**Figure 8:**
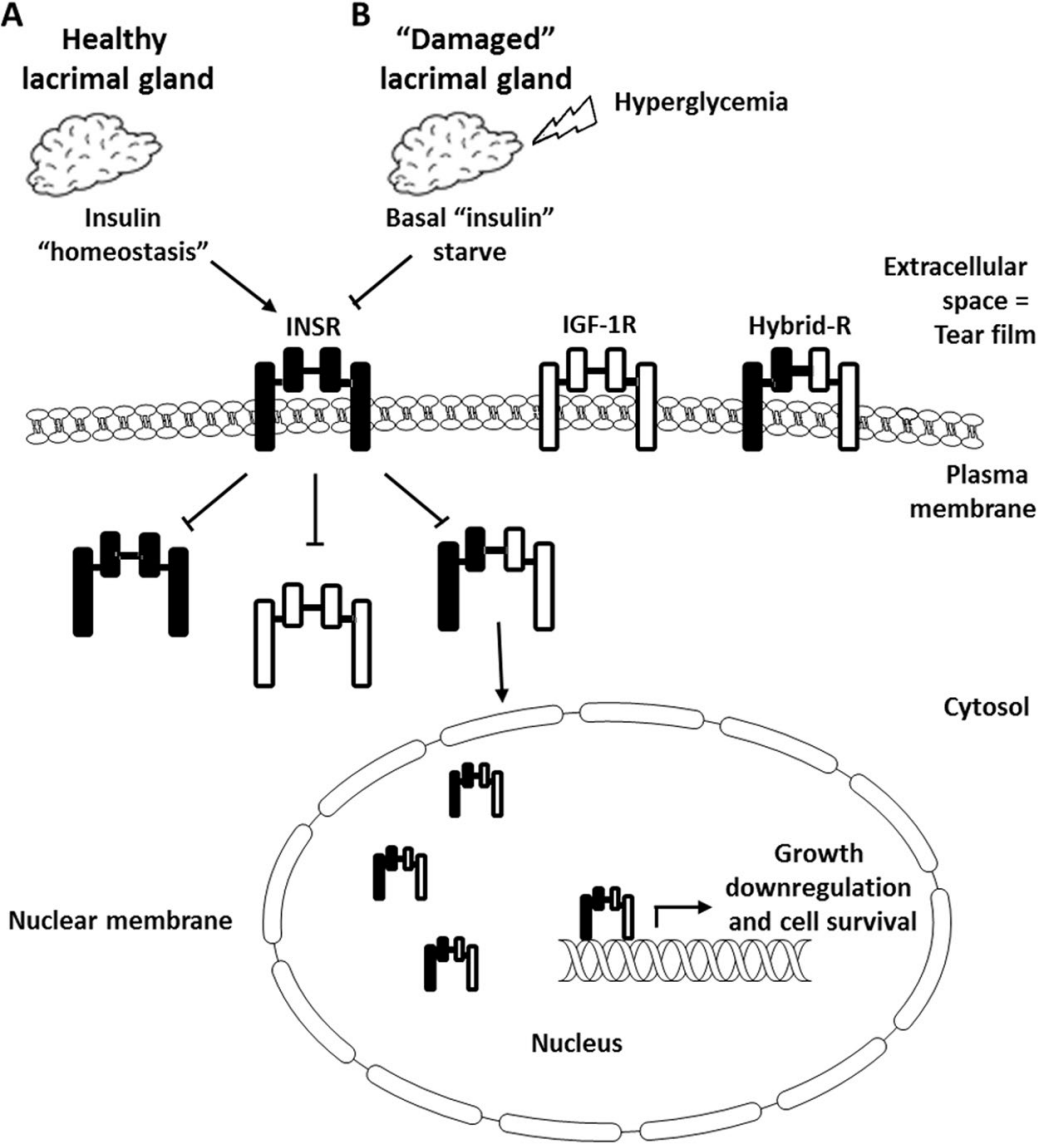
Schematic of the proposed hypothesis. (**A**) Insulin is secreted by the healthy lacrimal gland and maintains insulin homeostasis in the precorneal tear film. The presence of insulin downregulates INSR and IGF-1R through the homodimeric INSR to maintain the corneal epithelium. (**B**) In the presence of hyperglycemia, inflammation and oxidative stress, there is a reduction in insulin secretion as a result of a loss of corneal sensory nerves combined with direct damage to the lacrimal gland. The absence of adequate insulin levels results in an upregulation of INSR, IGF-1R and Hybrid-R and the subsequent accumulation of Hybrid-R in the corneal epithelial cell nucleus. In the nucleus, Hybrid-R directly binds DNA and functions to modulate the expression of genes involved in regulating cell growth and survival. Together, growth impairment and loss of neurotropic support leads to thinning of the corneal epithelium and abnormal wound healing.

In summary, extracellular insulin mediates Hybrid-R expression and *de novo* nuclear accumulation. Other receptor tyrosine kinases, including epidermal growth factor receptor (EGFR) have been shown to undergo ligand independent nuclear trafficking in response to DNA-damaging stress; however, this is the first report of ligand independent *de novo* Hybrid-R nuclear accumulation in any cell type^40,41^. This finding is unique to other tissues that require IGF-1 for induction of homodimeric IGF-1R nuclear trafficking^26^. Instead, IGF-1/insulin Hybrid-R expression is mediated through the homodimeric INSR in response to growth factor deprivation. This is associated with alterations in cell cycle control and mitochondrial homeostasis (Fig. 8). The corneal epithelium is subject to high physical, osmotic, glycemic, oxidative and inflammatory stress. Future studies will be aimed at determining whether this represents a global stress response in the corneal epithelium and whether this extends to other mucosal epithelial surfaces.

## Methods

### Cell lines and primary cultures

Human telomerized corneal epithelial cells (hTCEpi) were used in this study^42^. hTCEpi cells were cultured in serum-free keratinocyte basal media containing 0.15 mM calcium with supplements (KGM2, Lonza, Walkersville, MD) as previously reported^42^. In the growth deprivation condition, cells were cultured in keratinocyte basal media containing 0.15 mM calcium without supplements (KBM). For primary cultures (HCEC), human eye bank corneas (Tissue Transplant Services, UT Southwestern Medical Center, Dallas, TX) were digested in dispase (Invitrogen, Carlsbad, CA) overnight at 4 °C. Intact epithelial cell sheets were carefully removed and underwent a second digestion in dispase for 2 hours at 37 °C. Individual cells were separated by gentle pipetting and seeded onto plastic tissue culture dishes coated with Type 4 collagen (Biocoat, BD Biosciences, San Jose, CA). Cells were cultured in CnT20 cell culture media enriched for progenitor cell culture (Zen Bio, Research Triangle Park, NC).

### Reagents

Recombinant human insulin was purchased from Sigma (St. Louis, MO). For INSR neutralization, 5 µg/ml of a mouse monoclonal insulin receptor α antibody, clone MA-20 #sc-57344 and a mouse IgG control were purchased from Santa Cruz Biotechnology (Santa Cruz, CA). For PI3K inhibition, LY294002 from Millipore was used (Temecula, CA). Cycloheximide was purchased from Sigma (St. Louis, MO). The antibodies used for immunoblotting included: a rabbit polyclonal anti-IGF-1Rβ #3027 (130 µg/ml, 1:1000), a rabbit polyclonal phospho-IGF-IRβ (Tyr1131)/Insulin Receptorβ (Tyr1146) #3021(18 µg/ml, 1:1000) and a rabbit monoclonal anti-histone H3 #9715 (122 µg/ml 1:1000) (Cell Signaling, Danvers, MA), a mouse monoclonal anti-INSRβ #sc-57342 (200 ng/ml, 1:200), a mouse monoclonal anti-Akt1 #sc-5298 (200 ng/ml, 1:200), a rabbit polyclonal anti-phospho-serine 473 Akt1 #sc-7985-R (200 ng/ml, 1:200), a rabbit polyclonal GAPDH #sc-66163 (200 ng/ml, 1:200), and a rabbit IgG (Santa Cruz Biotechnology, Santa Cruz, CA), a mouse monoclonal anti-calnexin #MAB3126 (1 mg/ml, 1:500), a rabbit polyclonal anti-SP1 #ab13370 (1 mg/ml, 1:2000), and a mouse IgG (Millipore, Temecula, CA), and a mouse monoclonal anti-β-actin (Sigma, St. Louis, MO).

### siRNA knockdown of IGF-1R and INSR

For siRNA experiments, hTCEpi cells were seeded at 50–60% confluence in a 6-well tissue culture plate and grown overnight. Cells were transfected with double-stranded inhibitory RNA oligonucleotides (FlexiTube GeneSolution, INSR #GS3643 and IGF-1R #GS3480, Qiagen, Germantown, MD) using Lipofectamine RNAiMAX (Invitrogen, Carlsbad, CA) in antibiotic-free KBM. Briefly, 12 pmol of siRNA oligonucleotides were added to 100 µl KBM and incubated for 5 minutes at room temperature. siRNAs were then mixed with 2 µl Lipofectamine and allowed to incubate for an additional 20 minutes, after which the transfection mixture was added directly to hTCEpi cells containing 1 ml of KBM and incubated for 24 hours. Media was removed and cells were then cultured in KGM (growth) or KBM (basal) for another 24 hours as indicated. Allstars negative control siRNA was used as a non-targeting control (Qiagen, Germantown, MD).

### Real Time PCR

hTCEpi cells and HCECs were cultured in growth (KGM) and basal (KBM) conditions, the latter with and without 10 µg/ml insulin for 24 hours. RNA was extracted using an RNeasy kit (Qiagen, Germantown, MD) according to manufacturer instructions. Residual genomic DNA was removed using gDNA Wipeout Buffer (Qiagen, Germantown, MD). RNA concentration was determined using a Qubit 3.0 Fluorometer (ThermoFisher, Rockford, IL). 1 ug mRNA was reverse transcribed using a QuantiTect Reverse Transcription Kit (Qiagen, Germantown, MD). Real time PCR for IGF-1R and INSR was performed using a QuantiFast SYBR Green PCR kit (Qiagen, Germantown, MD). 100 ng of cDNA was amplified using QuantiTect Primer Assays (Qiagen, Germantown, MD) for Hs_INSR (QT02396128) and Hs_IGF-1R (QT00005831). 1 µM of each primer set was used in a total reaction volume of 25 µl and subject to 40 cycles of real time PCR. HS_β-actin (QT01680476) was used as a normalizing control in all experiments. Water was used as a no-template control. All controls were performed in parallel. For all experiments, samples were plated in triplicate and amplified using a QuantStudio 6 Flex Real Time PCR machine (Applied Biosystems, Foster City, CA). Experiments were repeated a minimum of two additional times. Data was analysed using the 2-ΔΔCT method.

### Subcellular fractionation and immunoblotting

To examine localization of IGF-1R and INSR in different cellular fractions, hTCEpi cells were subject to subcellular fractionation. Confluent hTCEpi cells were collected using trypsin-EDTA and washed with cold phosphate buffered saline (PBS). A high salt nuclear fractionation kit was used to separate nuclear and non-nuclear proteins (Thermo Fisher, Rockford, IL). Protein concentration for individual fractions was measured using a Bradford Assay (BioRad, Hercules, CA). 20 μl of protein from each fraction was loaded into individual lanes. Lysates were then electrophoresed through a 4–15% linear gradient precast polyacrylamide gel (BioRad, Hercules, CA) and immunoblotted as described below.

### SDS PAGE and Immunoblotting

hTCEpi cells or HCECs were lysed directly in 6-well culture plates using radioimmunoprecipitation (RIPA) buffer containing a protease and phosphatase inhibitor cocktail (Thermo Fisher, Rockford, IL) on ice for 10 minutes, then centrifuged for 5 minutes at 12,000 rpm at 4 °C in a microcentrifuge (BioRad, Hercules, CA). Protein concentration was determined using a Qubit 3.0 Fluorometer (Thermo Fisher, Rockford, IL). Supernatants were removed and boiled for 5 minutes in 2× sample buffer pH 6.8 containing 65.8 mM Tris-HCL, 26.3% (w/v) glycerol, 2.1% SDS, 5.0% β-mercaptoethanol and 0.01% bromophenol blue (Bio-rad, Hercules, CA). Samples were resolved on a 4–15% precast linear gradient polyacrylamide gel (Bio-rad, Hercules, CA) and transferred to a polyvinyl difluoride (PVDF) membrane (Millipore, Temecula, CA). Membranes were blocked in 5% non-fat milk (Bio-rad, Hercules, CA) for 1 hour at room temperature and incubated in primary antibody overnight at 4 °C. Following a 1 hour incubation with an anti-mouse or anti-rabbit secondary antibody (Santa Cruz, CA), protein was visualized using ECL Prime Detection Reagent (Amersham Biosciences, Piscataway, NJ) and imaged on an Amersham Imager 600 (Amersham Biosciences, Piscataway, NJ). For immunoblots of whole cell lysates, β-actin or GAPDH were used as loading controls.

### Immunoprecipitation

To test for the presence of the IGF/insulin hybrid receptor in hTCEpi cell nuclei, nuclear and non-nuclear fractions were immunoprecipitated with an anti-IGF-1Rβ monoclonal antibody (Cell Signaling, Danvers, MA, IGF-1Rβ #3027) or a rabbit IgG control. Two μl (260 ng) of antibody were incubated with 1 mg of protein from nuclear or non-nuclear fractions overnight at 4 °C with continuous rocking in a refrigerated environmental chamber (Thermo Fisher, Rockville, IL). Antibody-protein complexes were added to 50 μl immobilized protein A/G plus agarose (Thermo Fisher, Rockville, IL) for 2 hours at room temperature with rocking. Precipitates were washed with PBS and added to 25 μl 4 × sample buffer pH 6.8 containing 0.25 M tris, 8% lauryl sulfate, 40% glycerol, 20% mercaptoethanol and 0.04% bromophenol blue. Lysates were boiled for 5 minutes, followed by centrifugation for 3 minutes at high speed. 25 μl of supernatant was subject to SDS PAGE and immunoblotted as described above. Membranes were blotted for INSR and IGF-1R.

### Immunofluorescence

For immunofluorescence studies, hTCEpi cells were seeded onto 35 mm glass bottom dishes (MatTek Corporation, Ashland, MA) and allowed to adhere overnight. Cells were cultured in basal or growth media for 24 hours. After the treatments, cells were first rinsed with cold PBS and then fixed in 1% paraformaldehyde (Electron Microscopy Sciences, Fort Washington, PA) in PBS for 10 min. After 3 washes with PBS, cells were permeabilized in 0.1% Triton X-100 in PBS for 10 min and then blocked using 0.5% bovine serum albumin (Sigma, St. Louis, MO) in PBS for 30 min. Samples were then incubated in primary antibody against IGF-1R at 4 °C overnight and subsequently washed in PBS and stained with an Alexa Fluor 488 #4412 (Cell Signaling, Danvers, MA), secondary antibody for 1 h at RT. All samples were mounted on slides using Prolong gold antifade reagent with DAPI for the nuclei staining (Invitrogen, Carlsbad, CA) and imaged on a Leica SP2 laser scanning confocal microscope (Leica Microsystems, Heidelberg, Germany) using a 63× oil objective. All images were sequentially scanned to avoid spectral crosstalk between channels.

### Cell Cycle Assay

Propidium Iodide (PI)/RNase staining solution (Cell Signaling, Danvers, MA) was used for cell cycle analysis. hTCEpi cells were seeded into 6-well plates and cultured overnight in KGM. Media was removed and replaced with either growth or basal media with or without insulin stimulation for 24 hours. At the end of the incubation period, cells were trypsinized and centrifuged at 1500 rpm. Cells were then washed with phosphate buffered saline and fixed with cold ethanol for 15 minutes. Following fixation, cells were stained with PI for 40 minutes at 37 °C. The cell cycle was analyzed using a Cellometer K2 Fluorescent Viability Cell Counter (Nexcelom, Lawrence, MA). All assays were performed in triplicate and repeated a minimum of two additional times.

### Real time metabolic studies

Simultaneous measurement of cell oxygen consumption rate (OCR) and intracellular acidification rate (ECAR) was performed in real time using a Seahorse Metabolic Analyzer XFp (Agilent Technologies, Santa Clara, CA). hTCEpi cells were seeded into Seahorse XFp miniplates and cultured in growth and basal conditions with or without insulin stimulation at 37 °C, 5% CO_2_ for 24 hours. Before initiating measurements, cells were incubated for 1 hour at 37 °C with Seahorse XF base medium containing 1 mM pyruvate, 2 mM glutamine, and 10 mM glucose (pH 7.4) in a non-CO_2_ incubator. OCR and ECAR were analyzed using a Seahorse XFp Cell Energy Phenotype Test Kit (Agilent Technologies, Santa Clara, CA) according to manufacturer instructions. Ten μM oligomycin was added to inhibit ATP synthase at 18 minutes followed by three separate injections of 10 μM carbonyl cyanide 4-(trifluoromethoxy) phenylhydrazone (FCCP) at 20 minute intervals to alter the mitochondrial membrane potential and allow for maximal oxygen consumption due to uninhibited electron flow through the electron transport chain. Measurements were obtained every 6–7 minutes for a total of 94 minutes. Data were analyzed using the manufacturer provided Wave software, version 2.3.0. The ratio for OCR/ECAR was calculated for each experiment. All assays were performed in triplicate and repeated a minimum of two additional times.

### Statistical Analysis

All data are expressed as mean ± standard deviation. A student’s t-test was used for comparison between two groups. For comparison between three groups, a One-way ANOVA with appropriate post-hoc comparison test was used. For comparison between three groups with multiple factors, a Two-way ANOA with appropriate post-hoc comparison test was used. Statistical significance was set at P < 0.05.

## Electronic supplementary material

Dataset 1

Dataset 2
