# Supplementary material for: Insulin mediates de novo nuclear accumulation of the IGF-1/insulin Hybrid Receptor in corneal epithelial cells
Source: Sci Rep. 2018 Mar 12;8:4378. doi: 10.1038/s41598-018-21031-7 (PMC5847585; doi:10.1038/s41598-018-21031-7)

Insulin mediates *de novo* nuclear accumulation of the IGF-1/insulin Hybrid Receptor

in corneal epithelial cells

By

Rossella Titone, Meifang Zhu, and Danielle M. Robertson*

From the Department of Ophthalmology

The University of Texas Southwestern Medical Center

**Supplementary Figure 1:** Supraphysiological levels of insulin attenuate IGF-1R expression in corneal epithelial cells. hTCEpi cells were treated with increasing concentrations of insulin (862.1, 1724.1, 3448.3, and 4310.3 nmol/l corresponding to 5, 10, 20 and 25 ug/ml). IGF-1R and INSR expression in hTCEpi cells was increased in basal media and decreased upon addition of insulin at all concentrations tested.


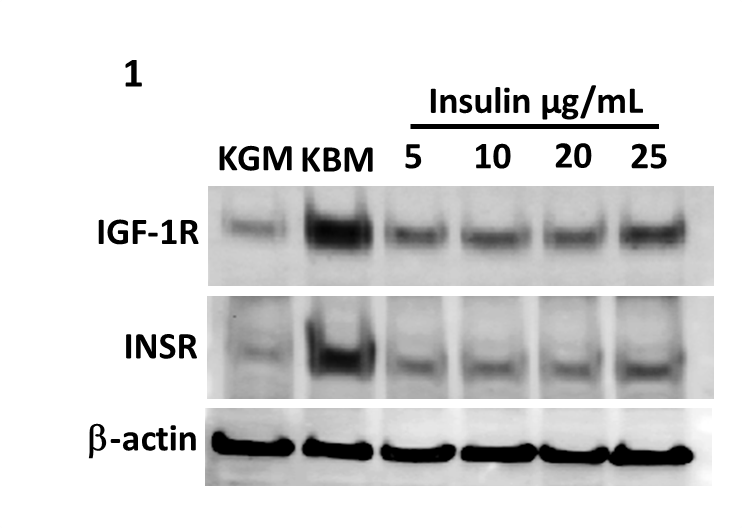


**Supplementary Figure 2:** Insulin in basal media induces phosphorylation of Akt in hTCEpi cells. Treatment of hTCEpi cells with the PI3 kinase inhibitor LY294002 blocked phosphorylation of Akt, but had no effect on IGF-1R or INSR expression in growth, basal and basal media supplemented with insulin.


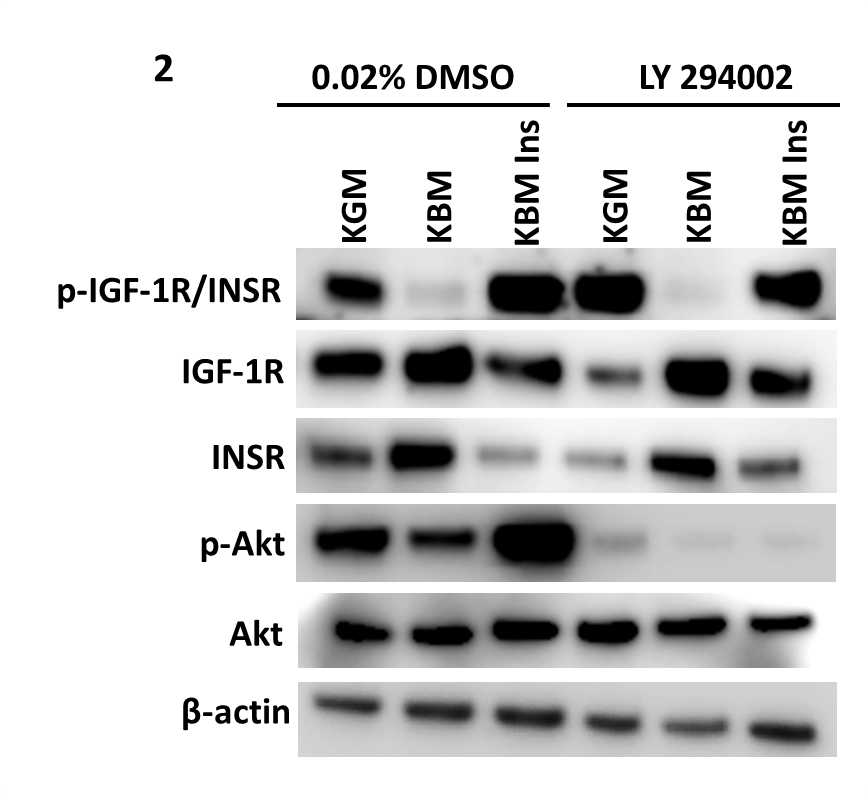

Supplement: Supplementary file 1 — Dataset 1 [file 41598_2018_21031_MOESM1_ESM.doc]
