# Supplementary material for: Insulin mediates de novo nuclear accumulation of the IGF-1/insulin Hybrid Receptor in corneal epithelial cells
Source: Sci Rep. 2018 Mar 12;8:4378. doi: 10.1038/s41598-018-21031-7 (PMC5847585; doi:10.1038/s41598-018-21031-7)

Insulin mediates *de novo* nuclear accumulation of the IGF-1/insulin Hybrid Receptor

in corneal epithelial cells

By

Rossella Titone, Meifang Zhu, and Danielle M. Robertson*

From the Department of Ophthalmology

The University of Texas Southwestern Medical Center

Figure S1: Whole membrane images for Figures 1A and 1B.


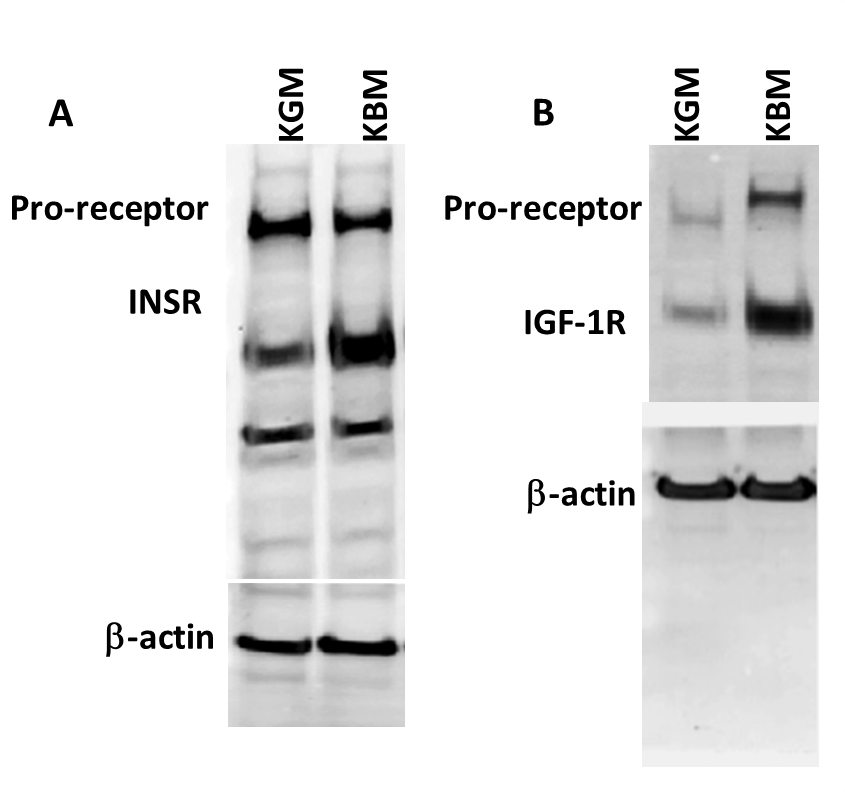


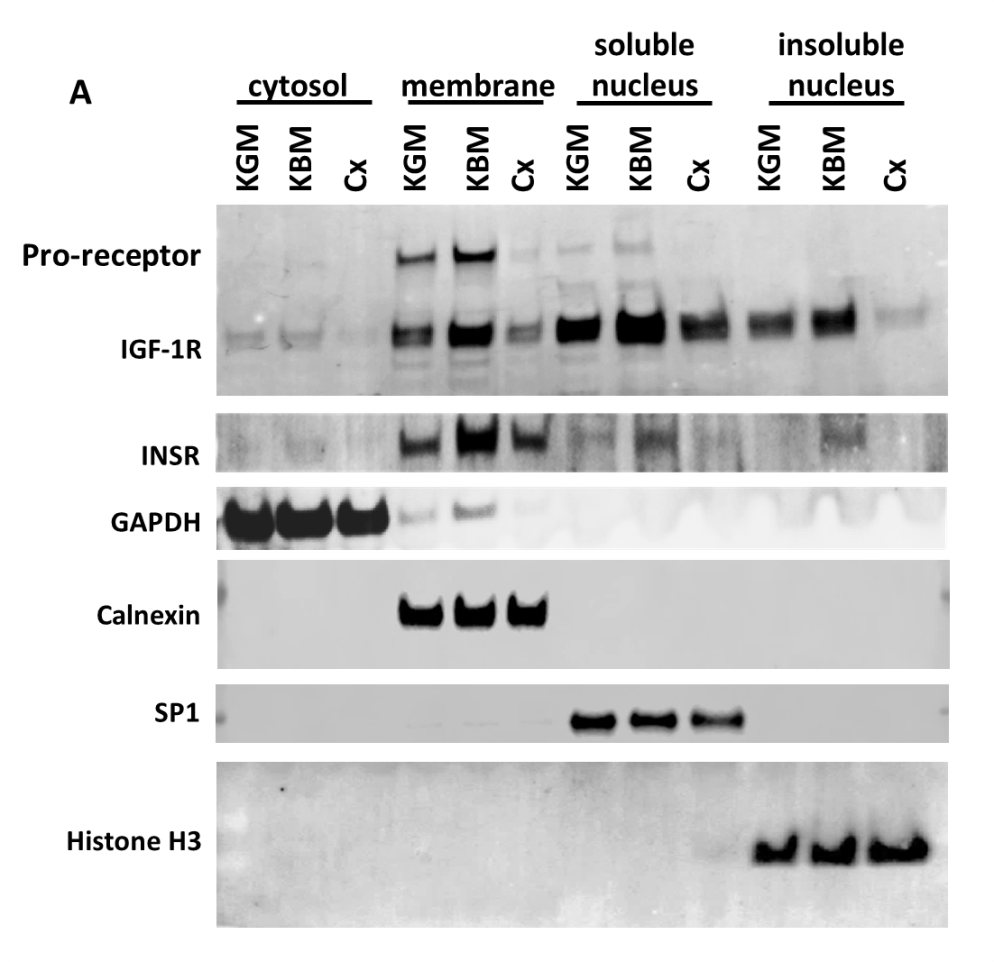
Figure S2: Whole membrane images for Figures 2A and 2B.


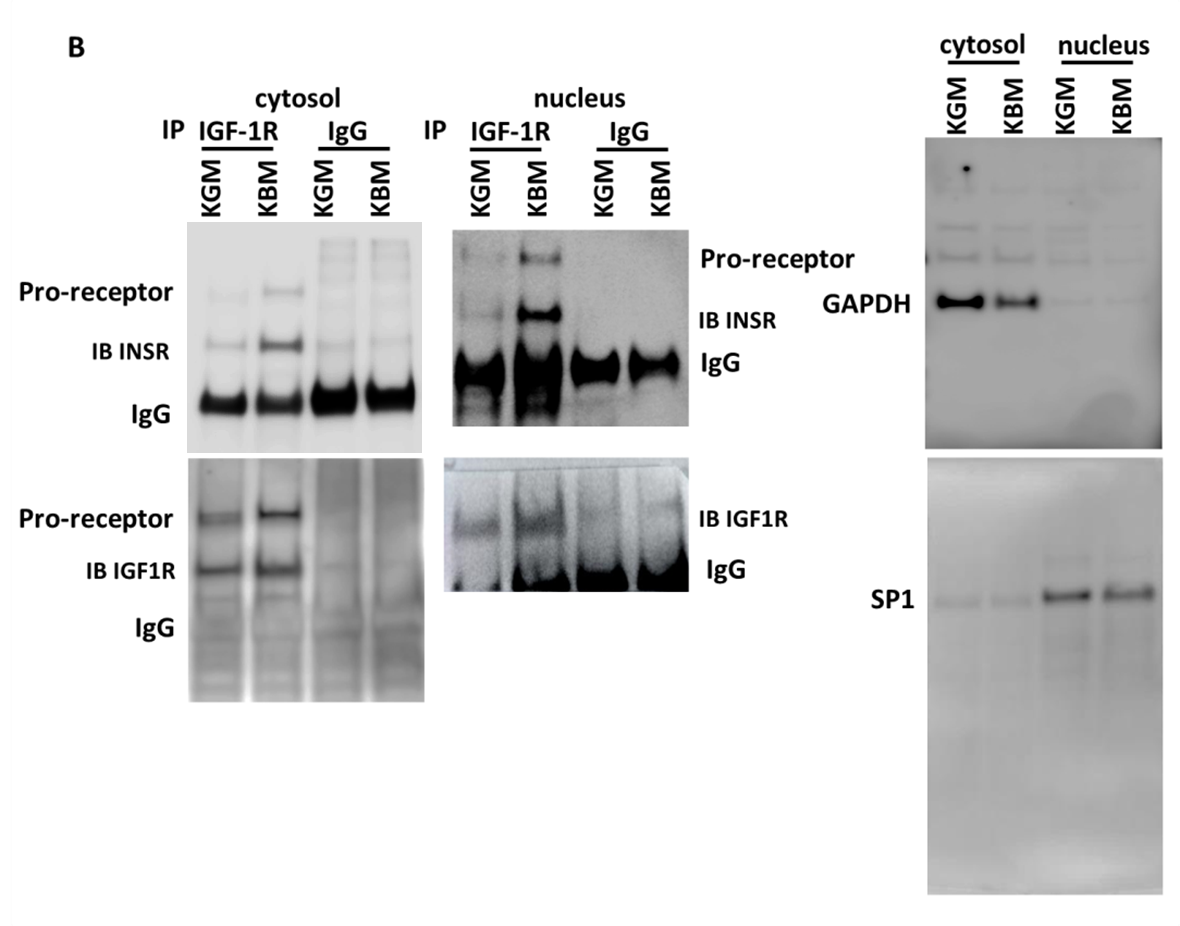


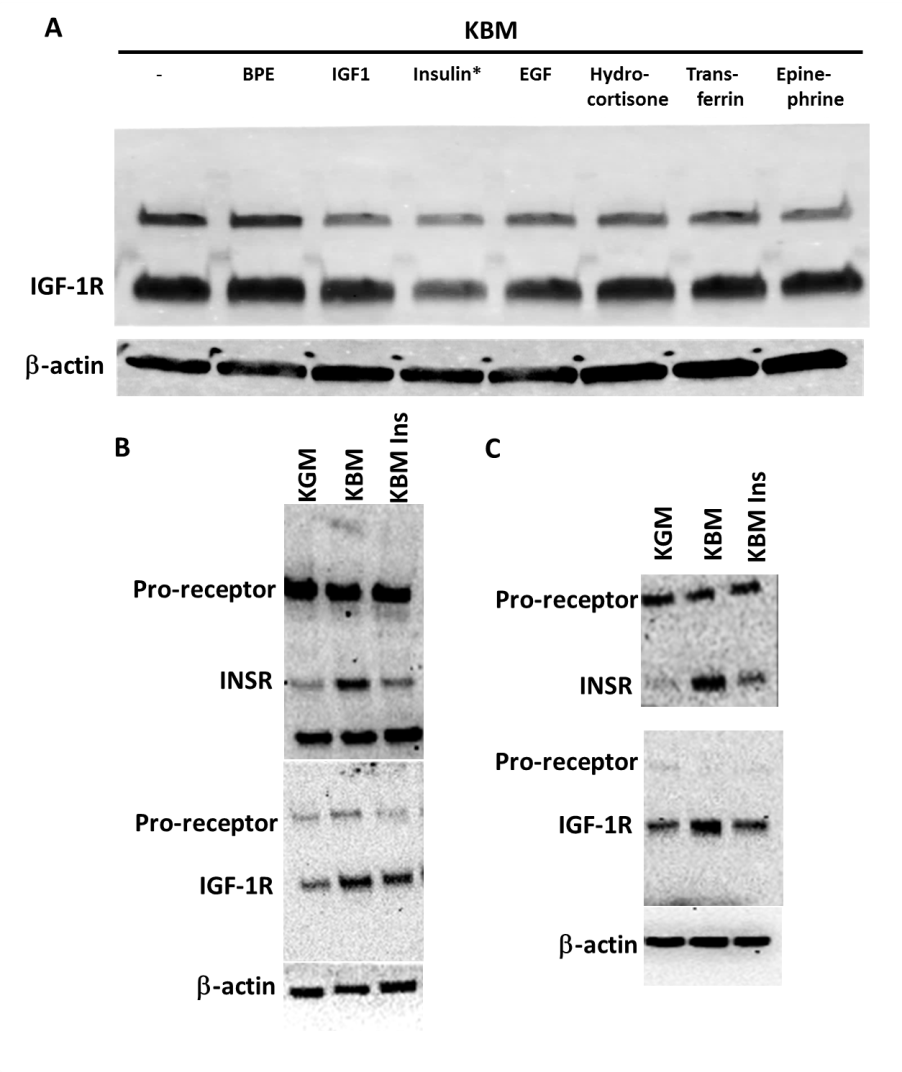
Figure S3: Whole membrane images for Figures 3A, 3B, and 3C.

Figure S4: Whole membrane images for Figures 4A and 4B.


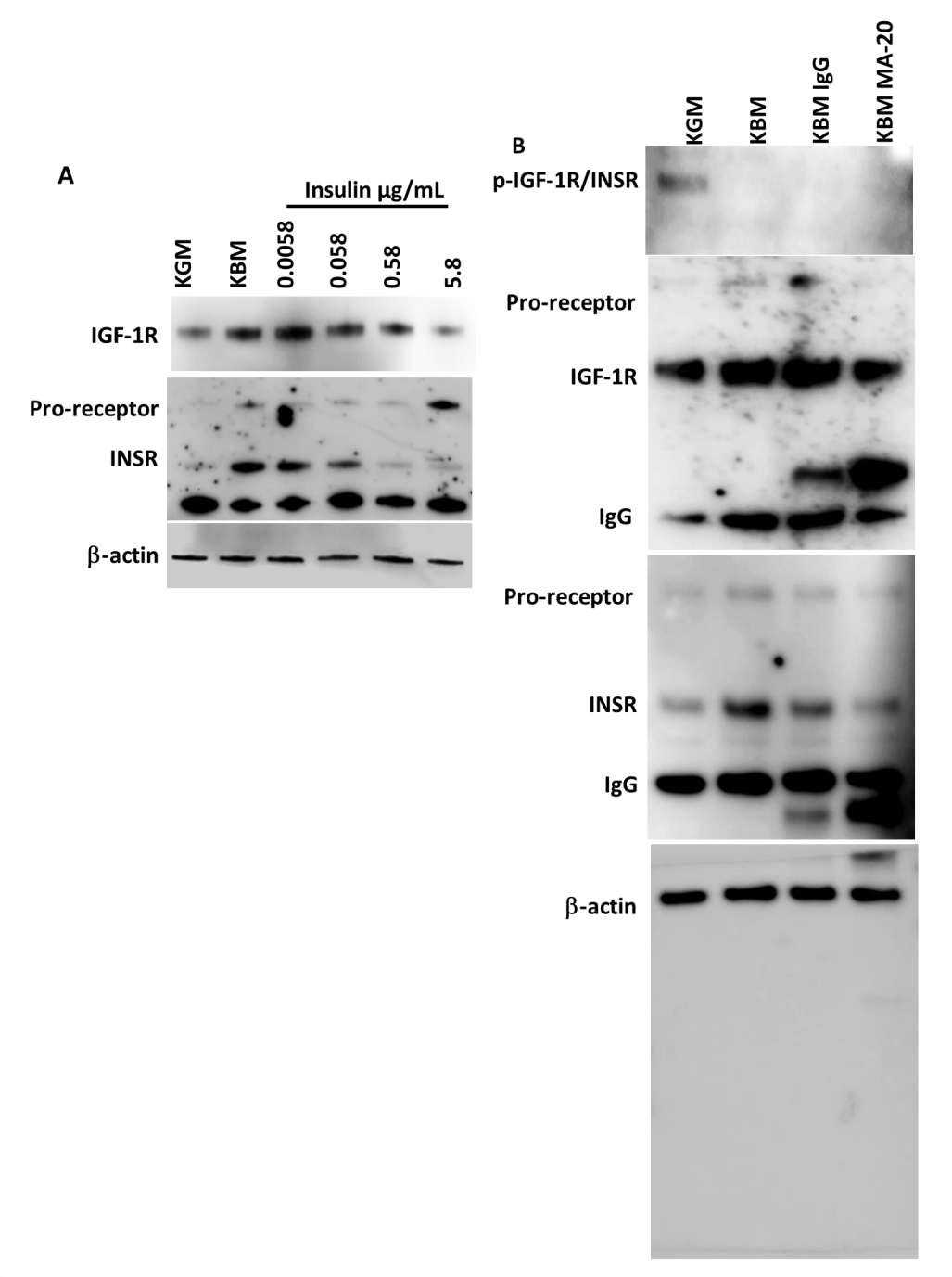


Figure S5: Whole membrane images for Figures 5A – 5D.


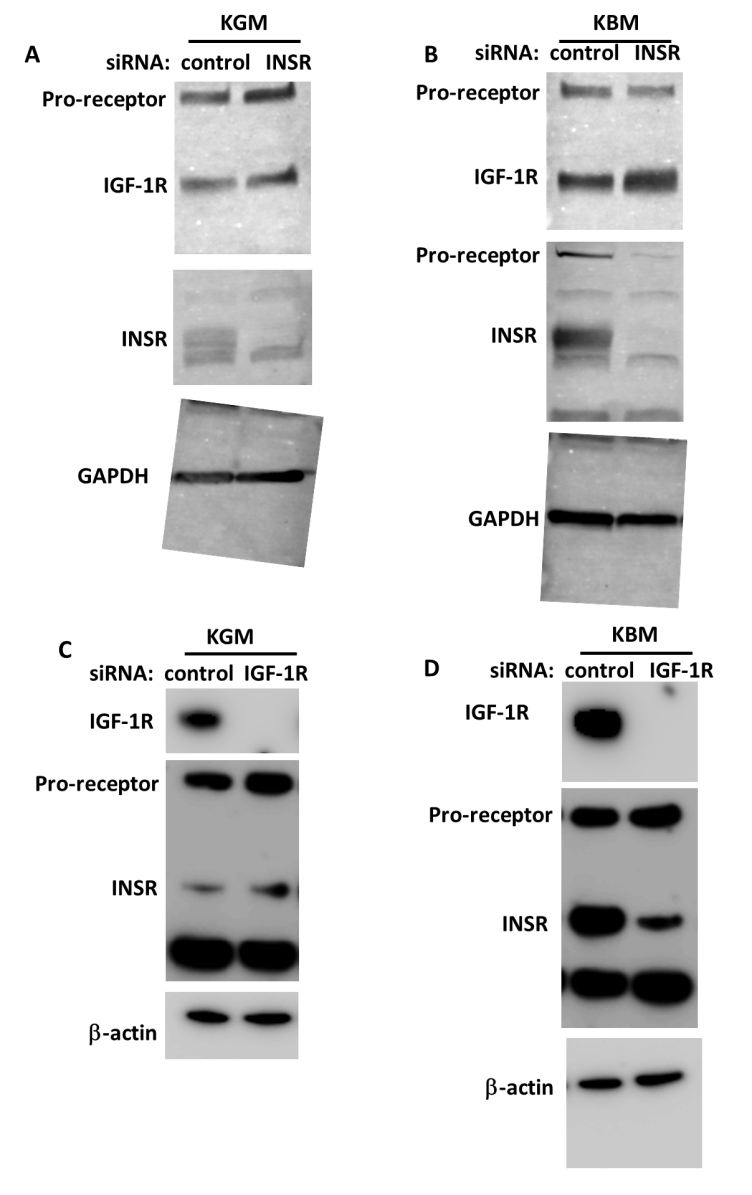


Supplementary dataset 1 whole membrane images.


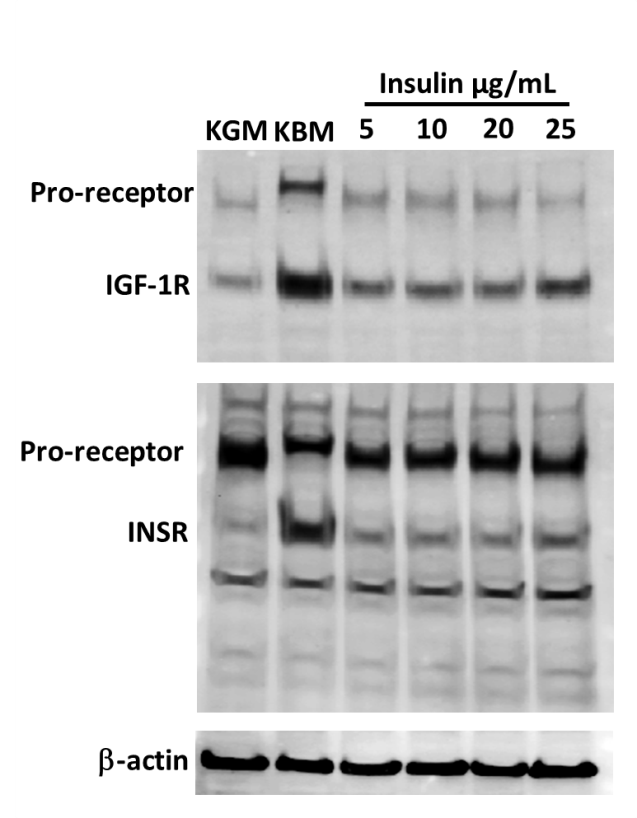
Supplementary Figure D1: Whole membrane image for supplementary figure 1.

Supplementary Figure D2: Whole membrane image for supplementary figure 2.


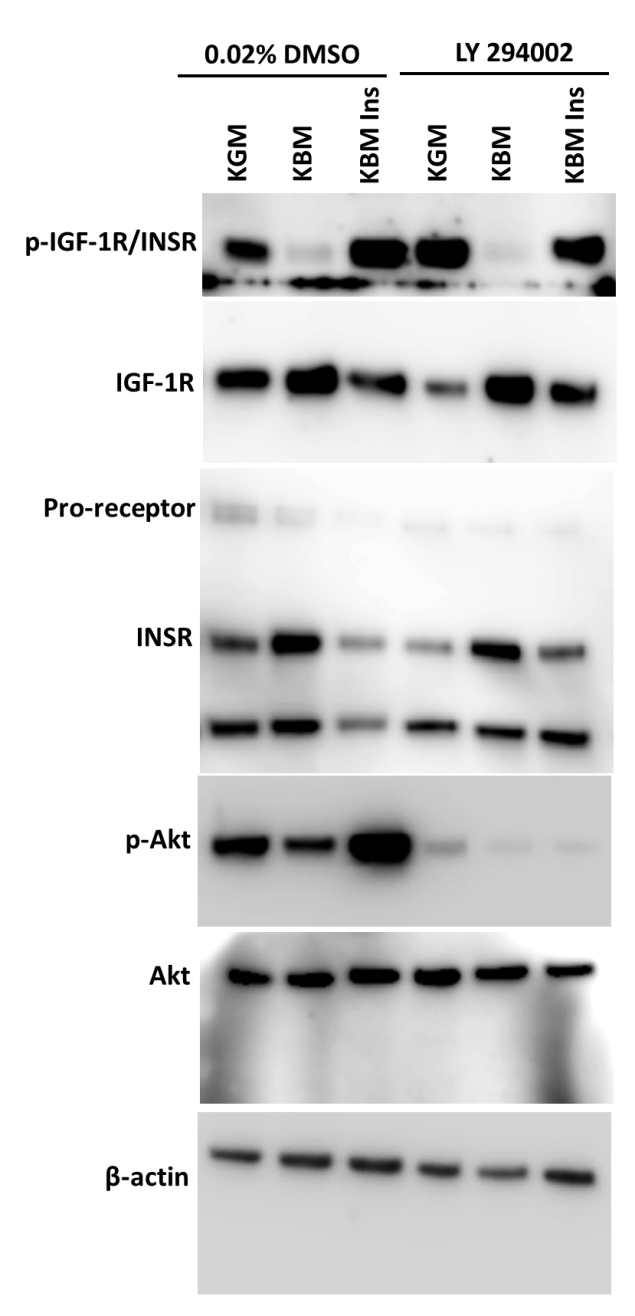

Supplement: Supplementary file 2 — Dataset 2 [file 41598_2018_21031_MOESM2_ESM.doc]
